# Position statement of the Microbiota International Clinical Society

**DOI:** 10.3389/frmbi.2025.1657750

**Published:** 2025-09-05

**Authors:** Chiara Maria Palazzi, Gaia Ciampaglia, Beatrice Binato, Mirko Ragazzini, Alexander Bertuccioli, Ilaria Cavecchia, Mariarosaria Matera, Massimiliano Cazzaniga, Giordano Bruno Zonzini, Nicola Zerbinati, Maria Laura Tanda, Francesco Di Pierro

**Affiliations:** ^1^ Microbiota International Clinical Society (MICS), Turin, Italy; ^2^ Department of Biomolecular Sciences (DISB), University of Urbino Carlo Bo, Urbino, Italy; ^3^ Microbiomic Department, Koelliker Hospital, Turin, Italy; ^4^ Department of Pediatric Emergencies, Misericordia Hospital, Grosseto, Italy; ^5^ Scientific Department, Velleja Research, Milan, Italy; ^6^ Department of Medicine and Technological Innovation, University of Insubria, Varese, Italy

**Keywords:** microbiota, gut microbiome, 16S rRNA gene sequencing, shotgun metagenomics, enterotypes

## Preamble

The intestinal microbiota is an extraordinarily complex ecosystem composed of trillions of microorganisms, including bacteria, viruses, fungi, and protozoa, which interact with each other and with the human host. This microbial community plays a crucial role in regulating human health, influencing vital processes such as digestion, immune modulation, inflammation control, and protection against pathogens (Di Pierro, 2019). Scientific and clinical interest inthe microbiota has grown exponentially in recent years, with implications ranging from metabolic and immune health to mental well-being (Hou et al., 2022). Microbiota analysis has emerged as a promising diagnostic and therapeutic resource for various clinical conditions. However, the lack of standardized regulations and limited concrete evidence of its clinical utility present significant challenges. Interest in the human microbiome has transformed biomedical research and clinical practice (Di Pierro et al., 2024a). The microbiome is now recognized as a key player in multiple biological processes, associated with conditions such as obesity, inflammatory bowel diseases (IBD), cardiovascular diseases, and neurological disorders (Porcari et al., 2025), thereby offering new opportunities for the development of novel therapeutic strategies as well as for complementing existing ones.

### Objectives of the position statement

This document aims to provide a comprehensive, state-of-the-art overview of the analytical, interpretative, and clinical requirements for microbiota testing, while also outlining current challenges, future opportunities, and operational guidelines based on the most recent scientific evidence.

The Microbiota International Clinical Society (MICS) aims to promote the development and a conscious, rational, appropriate, and standardized use of microbiota testing that can offer real benefits to clinical practice.

## The role of the microbiota in health and disease

The relationship between gut microbes and the human body is a mutualistic symbiosis, wherein the host provides a nutrient-rich environment that supports the microbiota, which in turn contributes to host well-being through the production of metabolites and other health-promoting substances, while simultaneously fostering the development of a functional immune system.

A growing body of scientific evidence highlights the fundamental role of the gut microbiota in maintaining host homeostasis (Adak and Khan, 2019) through the following functions:

**Metabolic functions:** The microbiota plays a crucial role in digestion, nutrient absorption, and the synthesis of essential vitamins, such as vitamin K and the B-vitamin complex [including biotin, cobalamin, folate, niacin, pantothenic acid, pyridoxine, riboflavin, and thiamine (Hill, 1997)]. It also contributes to the production of a broad range of metabolites, primarily derived from the bacterial fermentation of indigestible carbohydrates such as fibers and resistant starch.

Among the most relevant metabolites are short-chain fatty acids (SCFAs), including butyrate, propionate, and acetate, which exhibit key bioactive properties. These include the regulation of transepithelial fluid transport, enhancement of oxidative status, and reduction of mucosal inflammation. SCFAs also strengthen the intestinal barrier by increasing mucus production and enhancing the integrity of tight junctions, thereby impeding, for example, the progression of colorectal cancer (Weiss and Hennet, 2017). Butyrate, in particular, acts on the GPR109a receptor to stimulate the production of the cytoprotective cytokine IL-18 and promotes the differentiation of regulatory T cells (Treg) through the induction of IL-10, while inhibiting the formation of pro-inflammatory Th17 cells (Weiss and Hennet, 2017). They stimulate the release of anorexigenic peptides such as glucagon-like peptide- 1 (GLP-1) and peptide YY (PYY) from L-endocrine cells, contributing to satiety (Nicholson et al., 2012; Psichas et al., 2015).

**Structural functions:** Certain microbial species play a fundamental role in inducing the expression of proteins essential for tight junction function. They stimulate mucin production, a key component of the intestinal mucus barrier, and promote molecular signaling that supports epithelial cell survival, enhancing the organism’s ability to respond to inflammatory or infectious challenges (Ulluwishewa et al., 2011).

**Protective functions:** In a balanced intestinal ecosystem, specific microbial species can outcompete and suppress potentially pathogenic bacteria by producing antimicrobial compounds, altering intestinal pH, depriving pathogens of nutrients, and preserving the integrity of the mucosal barrier—an essential line of defense. Moreover, the microbiota modulates both innate and adaptive immune responses by influencing the production of inflammatory cytokines (Wu et al., 2017).

**Neurological functions:** The microbiota plays a key role in the gut-brain axis, a bidirectional communication system linking the gastrointestinal tract and the central nervous system (CNS). SCFAs stimulate enterochromaffin cells to produce and release serotonin, a neurotransmitter involved in mood regulation, memory, and learning (Carabotti et al., 2015).

## Eubiosis and dysbiosis

The human body hosts a complex ecosystem of microorganisms—primarily bacteria, but also fungi, viruses, and others. The gut microbiota (GM) plays a critical role in health and disease, as its activity influences digestive, metabolic, and immune functions. Once underestimated, the essential role of GM in physiological processes is now widely recognized (Qian et al., 2022). The human body consists of approximately 37.2 trillion cells, while the GM includes about 39 trillion microbial cells, highlighting a near 1:1 ratio. This balance emphasizes the symbiotic relationship between human and microbial cells. Found on our skin, in the mouth, respiratory tract, and especially the gut, the microbiota is a fundamental and indispensable ecosystem for human health and survival (Di Pierro, 2019; Shanahan et al., 2021). To describe the complexity of the symbiotic coexistence between a host organism, human, animal, or plant, and its associated biological entities that do not share the same DNA, the term “holobiont” is used. This concept extends to an integrated system that fulfills the biological needs defined by human DNA and the teleonomy of its ecosystem, including the GM. This paradigm shift also underscores the role of the mind in mediating between human needs and the microorganisms we host (Di Pierro, 2019).

Maintaining well-being inevitably requires the promotion of the teleonomy of specific bacterial taxa through dietary choices that favor their proliferation over microbes that may induce metabolic disturbances or dysbiosis (Fassarella et al., 2021). A eubiotic GM composition includes a balanced variety of bacterial, fungal, and viral taxa, which is crucial for health. Eubiosis refers to a harmonious and functional microbiota that supports the organism’s well-being. In contrast, dysbiosis is not merely an imbalance, but rather a microbial configuration that is misaligned with the host’s physiological needs, often influenced by genetic, dietary, and environmental factors. This condition may lead to reduced microbial diversity and promote the proliferation of pathogenic microorganisms, contributing to chronic inflammation.

In health, host–microbe symbiosis underpins digestive, metabolic and immune homeostasis, whereas dysbiosis—a departure from host-aligned community structure—has been associated with diverse pathologies spanning metabolic, inflammatory, oncologic and neuro-behavioral domains (Portincasa et al., 2022; Qian et al., 2022; Rusch et al., 2023; Wang et al., 2024). Four mechanistic axes recur across these associations: barrier dysfunction, including tight-junction impairment and mucus thinning; immune signaling driven by microbe- and diet-derived ligands that sustain low-grade inflammation (Turner, 2009; Weiss and Hennet, 2017); metabolic mediation via short-chain fatty acids and bile-acid derivatives that influence epithelial energetics and entero-endocrine output (Hill, 1997; Weiss and Hennet, 2017); and neuroactive routes, such as tryptophan/indole metabolism along the gut–brain axis (Carabotti et al., 2015; Zheng et al., 2016; Rusch et al., 2023). Because these links are largely correlative, we stress that cross-sectional taxonomic snapshots cannot establish causality; longitudinal multi-omics studies are essential to connect microbial function with clinical trajectories.

Another key aspect of dysbiosis is its impact on intestinal permeability. Alterations in the microbiota can compromise the integrity of the mucosal barrier, increasing intestinal permeability and promoting systemic inflammatory responses. Moreover, increased permeability may trigger neurogenic inflammation, crossing the blood-brain barrier and contributing to neuroinflammatory processes associated with conditions such as depression and anxiety (Rusch et al., 2023).

The gut microbiota and the intestinal mucosa, therefore, engage in a complex network of interactions that influence health under both physiological and pathological conditions. In a state of eubiosis, the mucus layer, rich in mucins, acts as a physical barrier against pathogenic colonization. Commensal bacteria also produce or stimulate the production of antimicrobial peptides and contribute to immune education (Turner, 2009).

In summary, the role and impact of the GM on human life are fundamental and indispensable. Understanding and maintaining a balanced microbiota through informed dietary and lifestyle choices can significantly support overall health and well-being (Rusch et al., 2023; Wang et al., 2024).

### Key factors influencing microbiota composition

- **Age:** The composition of the microbiota evolves with age. In newborns, it is less diverse and gradually develops through exposure to solid foods and environmental factors. The introduction of solid foods markedly increases ecological complexity, shifting the infant community from a *Bifidobacterium*-dominant profile toward an adult-like consortium dominated by Firmicutes and Bacteroidetes. Animal-protein and fat-rich weaning patterns often coincide with Bacteroides enrichment, whereas fiber-dense, carbohydrate-rich patterns favor Prevotella (Catassi et al., 2024; Mancabelli et al., 2024). Functionally, this transition elevates luminal SCFA production, lowers pH and primes mucosal immunity. Breastfeeding promotes the dominance of *Bifidobacterium*, supporting infant health, while formula-fed infants tend to have a more diverse but less stable microbiota, often enriched in *Clostridiales* and *Proteobacteria*. With the introduction of solid foods and increased dietary variety, the gut microbial community undergoes a significant shift, from a *Bifidobacterium*-dominated profile to one enriched in *Firmicutes* and *Bacteroidetes*, reflecting a more adult-like and functionally mature microbiota (Catassi et al., 2024). In adulthood, the microbiota generally reaches a stable state, although aging may then reduce microbial diversity and negatively affect overall health (Mancabelli et al., 2024).
- **Mode of delivery:** Infants born vaginally acquire microbiota like the maternal birth canal, supporting immune system development and a healthy adult microbiome. Those delivered via cesarean section are exposed to a more limited skin microbiota, typically dominated by *Staphylococcus* spp. and *Cutibacterium* spp., which may influence immune maturation and long-term health (Stewart et al., 2018).Breast milk also plays a critical role in microbiota maturation, containing immunological components and bioactive molecules, such as IgA, lysozyme, lactoferrin, and oligosaccharides, that promote the growth of beneficial bacteria like *Bifidobacteria* and *Lactobacilli*. Conversely, formula feeding tends to favor the proliferation of less beneficial intestinal bacteria (Gordon et al., 2012).
- **Sex:** Biological sex modulates microbial composition through intersecting mechanisms. Sex steroids—estrogen, progesterone and testosterone—alter growth and diversity of key taxa; higher estrogen correlates with increased Bacteroidetes diversity in women, whereas testosterone aligns with *Ruminococcus* and *Acinetobacter* enrichment in men (Dominianni et al., 2015; Graham et al., 2021; d’Afflitto et al., 2022; Hatayama et al., 2023). Differences in gastrointestinal transit time, mucosal immunity and urogenital anatomy further contribute to sex-specific community structures.
- **Host genetics:** Certain genetic variants may influence the immune system’s ability to interact with microorganisms, thereby shaping the composition of the microbiota (Hall et al., 2017).
- **Immune system:** A healthy immune system supports beneficial symbiosis between the host and the microbiota. Conversely, immune dysfunction, such as in autoimmune diseases, can disrupt this balance (Purchiaroni et al., 2013).
- **Health or disease status:** The presence of diseases, especially chronic or inflammatory conditions such as inflammatory bowel disease (IBD), diabetes, or cardiovascular diseases, can significantly alter the microbiota composition. Psychological conditions like stress and depression also negatively impact gut health (Iatcu et al., 2021; Quaglio et al., 2022; Liu L. et al., 2023).
- **Therapeutic drug use:** The use of antibiotics, nonsteroidal anti-inflammatory drugs (NSAIDs), and immunosuppressants profoundly affects the gut microbiota by reducing bacterial diversity and promoting the growth of resistant strains. At the same time, gut microorganisms produce enzymes capable of modifying drug efficacy and toxicity (Liu M. et al., 2023).
- **Diet:** Diet is among the most influential factors in shaping the microbiota. Diets rich in fiber—such as those based on fruits, vegetables, and whole grains—support the growth of beneficial bacteria and promote greater microbial diversity compared to meat-heavy diets (Moszak et al., 2020). Additionally, microbiota composition varies significantly based on geographical location and socioeconomic status, both of which influence dietary habits and lifestyle. For example, individuals living in urban environments tend to have less diverse microbiota than those in rural areas, often due to a less varied diet and reduced microbial exposure (Ayeni et al., 2018). A recent study examined how different foods and cooking methods modulate the gut community using *in vitro* digestion– fermentation models inoculated with human fecal microbiota (Hatayama et al., 2023). The study found significant differences in both composition and diversity. For instance, using fats, particularly butter, as substrates increased the abundance of potentially beneficial taxa such as *Faecalibacterium*, *Roseburia*, and *Blautia*. Frying and grilling tended to reduce *Ruminococcaceae*, whereas boiling appeared to decrease *Firmicutes*. However, while general trends emerged, individual variability significantly affected the results, complicating the generalization of findings (Hatayama et al., 2023).

## Microbial biodiversity

Much of the current knowledge about the human microbiota comes from the Human Microbiome Project (HMP), a major initiative that mapped and characterized the healthy microbiome in various body regions including the gut, skin, mouth, nose, and vagina. The HMP also explored the microbiome’s role in numerous conditions such as obesity, diabetes, gastrointestinal disorders, autoimmune diseases, and neuropsychiatric disorders—laying the foundation for new medical and biotechnological research.

In the first phase of the project, the HMP revealed that everyone possesses a unique microbiome with site-specific microbial specialization. Its composition is influenced by factors such as diet, genetics, and environment (Turnbaugh et al., 2007).

The second phase, the Integrative Human Microbiome Project (iHMP), confirmed the link between the microbiome and chronic diseases, showing that a loss of microbial diversity is associated with increased risk of such conditions (The Integrative et al., 2019).

Despite the significant interindividual variability in gut microbiota revealed by the HMP, some common features exist. The gut microbiota comprises over 1,500 species across more than 50 phyla (Robles-Alonso y Francisco Guarner, 2013). Of these, *Bacteroidetes* and *Firmicutes* are the most dominant, followed by *Proteobacteria*, *Fusobacteria*, *Tenericutes*, *Actinobacteria*, and *Verrucomicrobia*, which together account for up to 90% of the total microbial population in humans (Jethwani and Grover, 2019).

Within the Firmicutes phylum, there are approximately 200 different genera, including *Bacillus*, *Lactobacillus*, *Enterococcus*, *Clostridium*, and *Ruminococcus*. While *Lactobacillus* species are known for their health-promoting effects, certain *Firmicutes*— such as *Staphylococcus aureus* and *Clostridium perfringens*—can be pathogenic when they proliferate excessively. Among the *Bacteroidetes*, *Bacteroides* and *Prevotella* are the predominant genera. The *Actinobacteria* phylum is less abundant and primarily represented by *Bifidobacterium*, known for its beneficial impact on health. Finally, *Proteobacteria* includes several well-known pathogens such as *Enterobacter*, *Shigella*, *Salmonella*, and *Escherichia coli* (MetaHIT Consortium (additional members) et al., 2011).

Biodiversity is a central concept in microbiology and microbial ecology, referring to the richness and distribution of species within a complex ecosystem such as the gut. Understanding microbial biodiversity in the gut is a key parameter for assessing the health status of the host.

Microbial biodiversity analysis helps identify dysbiotic states potentially linked to various pathologies (Turnbaugh et al., 2007). Detecting such alterations is critical to designing targeted therapeutic strategies aimed at restoring microbiota balance and improving host health.

This assessment relies on the identification of Operational Taxonomic Units (OTUs), a practical way to group microorganisms based on genetic similarity. Conventionally, an OTU includes all reads (genetic sequences, typically from the V3-V4 regions of the 16S rRNA gene) from a sample that share at least 97% similarity, a threshold widely adopted by the scientific community for taxonomic classification (Nguyen et al., 2016).

The 16S rRNA gene is a gold-standard phylogenetic marker in microbial taxonomy due to its ubiquity among bacteria and its combination of conserved and hypervariable regions. The conserved regions allow for universal primer binding, while the hypervariable regions, particularly V3-V4, enable effective discrimination between bacterial taxa while remaining compatible with next-generation sequencing platforms (Petti et al., 2005; López-Aladid et al., 2023).

Identification and quantification of OTUs provide critical information on the structure and composition of the gut microbiota, enabling comparisons across different experimental and clinical conditions (Ngom-Bru and Barretto, 2012).

To quantify intestinal microbiota biodiversity, two primary metrics are used (Sala et al., 2020):

- **Alpha diversity:** Measures the variety of species within a single gut sample. Common indices include the Shannon Index (Shannon, 1948), which evaluates both species richness and relative abundance, and the Chao1 Index (Chao, 1984), which estimates the actual number of species by accounting for rare taxa.
- **Beta diversity:** Compares microbial diversity between individuals or across different health conditions, revealing variations in microbial composition.

For accurate evaluation of these metrics, tools such as the rarefaction curve are employed. This curve depicts the relationship between the number of reads and the number of OTUs identified in the sample (Colwell and Coddington, 1994). In recent years, advanced software for metagenomic analysis has been developed to process sequencing data from raw reads to interpretation and database deposition. Among these, QIIME (Quantitative Insights Into Microbial Ecology) is one of the most widely used platforms for analyzing high-throughput sequencing data from microbiome studies. QIIME enables sequence clustering, taxonomic classification, and generation of rarefaction curves, facilitating the study of microbial composition across samples (Caporaso et al., 2010).

Comparing the rarefaction curve of a sample with that of a reference standard—whether from a healthy individual or an experimental control—enables the evaluation of sequencing depth (i.e., the number of reads) and the adequacy of microbial diversity representation. This provides fundamental insights into the stability and resilience of the gut microbiota under different physiological or pathological conditions. Therefore, analyzing rarefaction curves helps determine whether microbial biodiversity that is too low or excessively high compared to reference standards may indicate an altered microbiota status.

Low biodiversity has been associated with diseases such as atopy (Haahtela et al., 2013), autoimmunity (Wei et al., 2020), metabolic disorders (Wilmanski et al., 2019), inflammatory bowel disease (Mancabelli et al., 2017a), and *Clostridium difficile* infections (Schubert et al., 2014; Gazzola et al., 2020). Conversely, high biodiversity has been linked to conditions such as constipation, IBS-C (Irritable Bowel Syndrome, constipation-predominant), SIBO (Small Intestinal Bacterial Overgrowth) and neurological disorders associated with constipation (Mancabelli et al., 2017b).

Alpha diversity of the intestinal microbiome is increasingly recognized by the scientific community as a potential predictive indicator of various diseases. One research group studied the impact of antibiotic use combined with influenza vaccination, finding that the loss of bacterial functionality due to antibiotics weakened immune responses and reduced vaccine efficacy (Hagan et al., 2019). Additionally, low gut biodiversity has been linked to immunosuppression, which impairs the immune system’s ability to suppress tumor growth.

This condition has been associated with significantly lower survival rates in patients with pancreatic cancer, suggesting that a healthy and diverse gut microbiome may play a crucial role in supporting immune responses against cancer (Pushalkar et al., 2018).

In this context, the evaluation of biodiversity, through parameters such as alpha and beta diversity and rarefaction curves, becomes a critical tool for designing personalized interventions and optimizing clinical treatments.

## Enterotypes

Over time, various modeling frameworks have been proposed in the literature to classify human enterotypes. Initial models identified two opposing groups: *Bacteroides + Firmicutes* and *Prevotella*. This classification later evolved into three distinct groups: *Bacteroides*, *Prevotella*, and *Ruminococcus* (the latter representing *Firmicutes*). A further refinement led to four categories: *Bacteroides*, *Prevotella*, Mixed Type 1 (*Firmicutes + Bacteroides*), and Mixed Type 2 (*Firmicutes + Prevotella*) (Di Pierro, 2021).

Human gut enterotypes are not associated with sex, age, or body weight but are influenced by long-term dietary habits (Liang et al., 2017). Based on current literature, the three majors human enterotypes can be defined as follows:

1. **Prevotella Enterotype (ET-P):** associated with diets rich in carbohydrates.
2. **Bacteroides Enterotype (ET-R):** typical of balanced, fiber-rich diets.
3. **Firmicutes Enterotype (ET-B):** commonly found in high-fat, high-protein diets.

**Figure 1:**
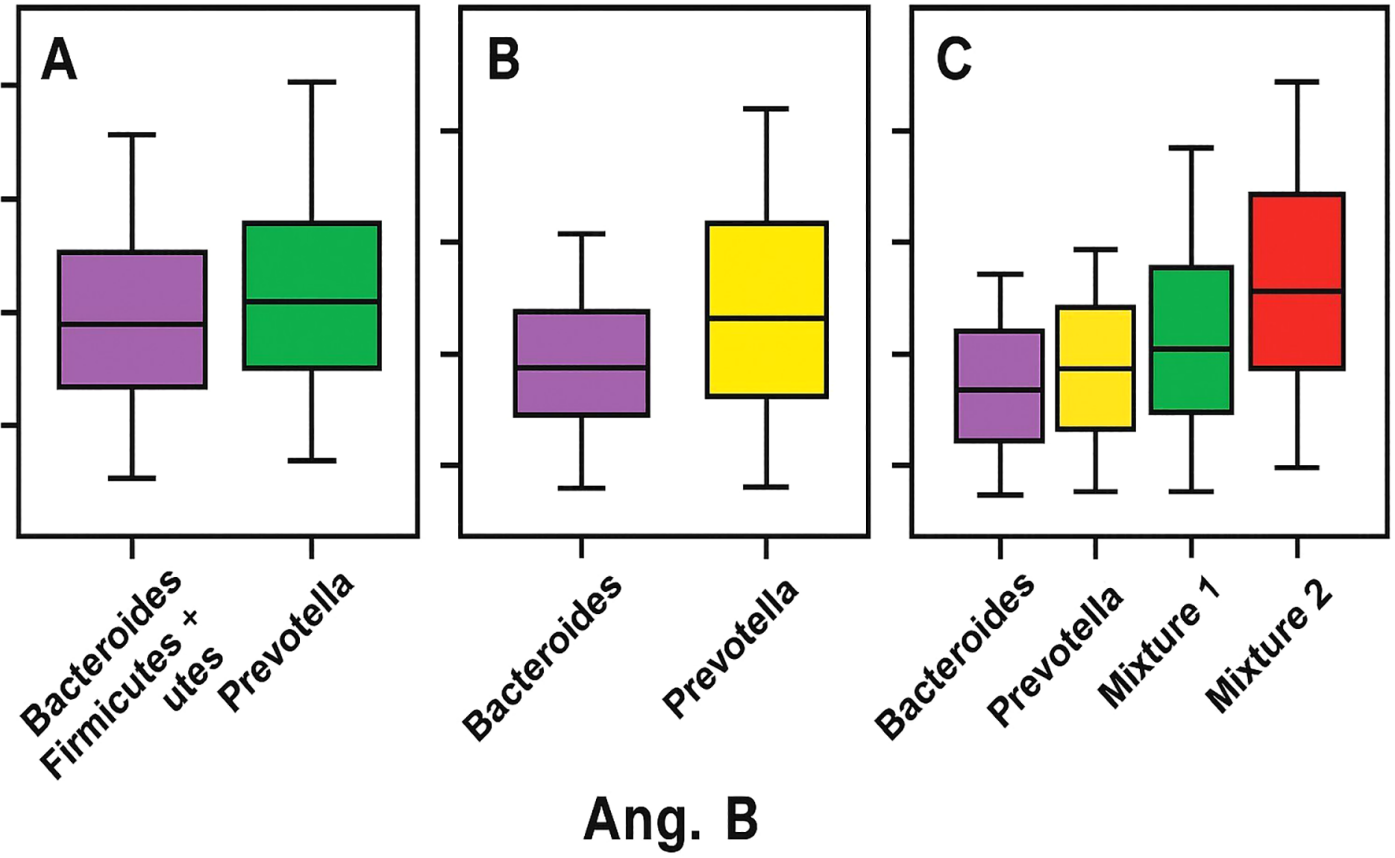
Enterotype-related patterns in adult stool metagenomes. **(A)** Relative abundances of *Bacteroides and Prevotella*. **(B)** Functional richness across enterotype clusters (*Bacteroides*-dominant, *Prevotella*-dominant, and mixed profiles), with higher predicted functional capacity in *Prevotella*-dominant and Firmicutes-mixed profiles. **(C)** Mixed profiles (*Bacteroides+Prevotella* and *Prevotella+Bacteroides*) illustrating increasing dispersion.

In addition, mixed enterotypes, Firmicutes + Bacteroidetes (ET-M1) and Firmicutes + Prevotella (ET-M2), represent combinations of the respective microbial characteristics. These distinct clusters are based on the predominance of specific bacterial genera and are closely linked to long-term dietary patterns rather than factors such as age, sex, or geographic origin. This finding underscores the significant impact of diet on gut microbiota composition (Wu et al., 2011). As illustrated in **Figure 1** Ang. B, the graph depicts the relationship between gut microbiota functional richness (Y-axis) and distinct microbiota clusters or enterotypes (X-axis), as defined by compositional models considering two, three, or four dominant configurations. The X-axis represents taxonomic clusters based on the dominance of specific bacterial genera, primarily *Bacteroides*, *Prevotella*, and *Ruminococcus*, or composite profiles (e.g., Mixture 1 and 2). The Y-axis refers to functional richness, reflecting the predicted metabolic diversity of the microbial community rather than taxonomic richness (e.g., OTUs or ASVs). The graph shows that enterotypes dominated by *Prevotella* or by Firmicutes (Mixture 2) exhibit higher functional richness compared to *Bacteroides*-dominated profiles, suggesting that enterotype structure may influence the functional capacity of the gut microbiome (Di Pierro, 2021). Functional richness refers to predicted metabolic capacity, not to taxonomic counts, and therefore must be interpreted cautiously.

Everyone, therefore, possesses a unique “microbial signature,” shaped by genetic, dietary, environmental, and lifestyle factors. This makes the gut microbiota a key indicator of personal health and opens the door to potential diagnostic and therapeutic applications.

The various enterotypes differ not only in the predominant bacterial taxa but also in their functional characteristics and behavior as microbial ecosystems. For instance, Prevotella- dominated enterotypes, typically associated with carbohydrate-rich diets, tend to be more diverse and abundant, resulting in a more resilient and stable microbial structure over time. In contrast, Bacteroides-dominated enterotypes are often characterized by lower richness and a higher concentration of dominant species. These differences influence not only the composition of the microbiota but also its interactions with the host and its overall impact on health (Gorvitovskaia et al., 2016).

Enterotype classification paves the way for personalized nutritional and therapeutic interventions based on an individual’s unique microbial signature (Costea et al., 2018). Understanding and mapping these configurations could provide clinicians and nutritionists with more comprehensive diagnostic tools, enabling a more individualized approach to patient care. Although scientific and clinical interest in gut microbiota is rapidly growing, research in this area remains in its early stages. Nevertheless, some practical applications are already emerging, such as fecal microbiota transplantation (FMT), a well-established and recommended therapy for recurrent *Clostridioides difficile* infections (Liubakka and Vaughn, 2016). These advancements point toward a future in which microbiota analysis and modulation could revolutionize clinical practice, enabling increasingly personalized therapeutic strategies for a wide range of diseases.

Microbiota testing enables characterization of the gut microbial composition, making a valuable contribution to the prevention, monitoring, and treatment of diseases in a highly personalized manner. This approach supports the goals of precision medicine by identifying microbial imbalances, such as dysbiosis, which may impair host health and predispose individuals to specific pathological conditions. Tailoring interventions to an individual’s microbiota profile has the potential to optimize health outcomes by delivering targeted and customized therapeutic strategies (Bender et al., 2023).

It is essential to emphasize, however, that despite growing enthusiasm for the clinical role of the gut microbiota, current scientific evidence remains limited. At present, most clinical applications are based on preliminary studies or highly specific contexts, and there is not yet sufficient evidence to support the widespread use of microbiota testing or interventions in routine clinical practice (Di Pierro et al., 2024a).

## Requirements and standardization of microbiota testing

The prescription of a microbiota test requires a careful and well-informed assessment of the clinical rationale justifying its execution. This decision must be based on a thorough analysis of the patient’s medical history, current symptoms, and overall health status to ensure that the test is truly beneficial for diagnostic or therapeutic purposes. For this reason, microbiota testing should only be requested by physicians or other qualified healthcare professionals with the necessary expertise to correctly interpret the results and integrate them into the patient’s clinical management.

However, the clinical adoption of microbiome testing is currently hindered by:

- A lack of standardization in analytical methodologies.
- The proliferation of commercial clinical tests with limited or no proven clinical utility.
- Difficulties in interpretation and a lack of education among healthcare professionals.

Statistical data indicate that microbiota analysis is primarily sought by biologically female individuals suffering from gastrointestinal disorders, typically functional in nature, characterized by bloating and abdominal distension (Chey et al., 2015). These tests also attract healthy individuals curious about their microbiota, as well as patients affected by various conditions, most commonly gastrointestinal, gynecological, or other systemic disorders. Nonetheless, interest in these tests is mainly driven by non-medical individuals, particularly patients themselves. Many of these individuals are likely searching for answers to long-standing medical concerns that conventional healthcare has failed to adequately address—answers that have not been perceived as either “complete” or “satisfactory”.

The value of direct-to-consumer microbiota analysis has recently come under strong criticism. According to several authors, such testing does not meet the three fundamental criteria that any clinical test should fulfill:

1. **Analytical validity**: The ability to accurately measure the microbiome.
2. **Clinical validity**: Scientific evidence linking microbiome profiles to specific health conditions.
3. **Clinical utility**: The test’s capacity to positively influence patient management.

Analytical validity ensures that the test can reliably detect and quantify the intended components.

Clinical validity refers to the test’s ability to determine the presence of a specific disease. Clinical utility is defined as the test’s ability to guide therapeutic decisions (Di Pierro et al., 2024a).

## Analytical validity

The analytical validity of microbiota testing is essential to ensure accurate and reproducible results. This concept refers to the test’s ability to consistently measure what it is intended to detect, with minimal error. To achieve this, tests must be accurate, precise, and sensitive. However, in the context of microbiota analysis, fully meeting these criteria is challenging due to the intrinsic variability of microbial composition and the complexity of analytical methods. Nevertheless, a high-quality microbiota test should strive to provide results that are as reliable as possible.

Key requirements to achieve this goal include:

- **Sample collection and preservation:** Samples must be collected using specially designed kits containing chemical preservatives, such as alcohol-based solutions or other stabilizers, to maintain bacterial DNA integrity during transport and storage. These procedures require specific validation depending on whether the sample is fecal or vaginal.
- **Standardized storage:** Preservation at 80 °C is recommended to prevent microbial degradation prior to analysis. (Note: clarify specific implications with Di Pierro).
- **Environmental decontamination:** Strict procedures must be implemented to prevent contamination during collection and transportation.

If analytical validity is achieved, or at least reaches a compromise with an acceptable level of error, then the issue posed by direct-to-consumer microbiota testing could, at least temporarily, be mitigated by restricting analyses to laboratories whose analytical capabilities and methods are recognized by the scientific literature. Providers of such consumer-directed microbiota tests should be required to clearly specify protocols for standardized sample collection and methodological accuracy (Porcari et al., 2025).

### 16S rRNA sequencing

The 16S ribosomal RNA (rRNA) gene sequencing method has long been considered the gold standard for taxonomic and phylogenetic characterization of bacterial communities in microbiota research (Clarridge, 2004). This technique targets the 16S rRNA gene, a highly conserved genetic marker universally present in prokaryotic organisms. Spanning approximately 1,500 base pairs, the 16S rRNA gene comprises both conserved and hypervariable regions. The conserved regions serve as binding sites for universal primers, facilitating the amplification of the gene across a wide range of bacterial species, while the hypervariable regions (e.g., V3–V4 or V4) allow for the differentiation and identification of bacteria at various taxonomic levels, including genus and species.

In gut microbiota research, 16S rRNA sequencing remains one of the most widely used methods due to its efficiency, accuracy, and scalability in profiling complex microbial ecosystems. Unlike traditional culture-based techniques, which can significantly bias results by capturing only a small fraction (often less than 1%) of the total microbial community, 16S rRNA sequencing bypasses this limitation by directly analyzing bacterial RNA, providing a comprehensive overview of microbial diversity.

Advantages of 16S rRNA sequencing include cost-effectiveness, rapid turnaround times, and the ability to detect a broad range of bacterial taxa, including those that are difficult or impossible to culture. Additionally, the method benefits from well-established bioinformatic workflows and reference databases for taxonomic assignment, making it ideal for generating broad taxonomic profiles and valuable snapshots of microbial diversity and abundance (Chakravorty et al., 2007).

Despite its wide application, 16S rRNA sequencing presents intrinsic limitations. One major drawback is its relatively low taxonomic resolution compared to whole genome sequencing (WGS). While 16S rRNA sequencing can reliably classify bacteria at the genus level, it often struggles to distinguish closely related species, particularly within highly diverse bacterial families. Moreover, it does not provide direct functional insights into the microbial community, as it targets a single gene rather than the entire genome (Muhamad Rizal et al., 2020).

The standard workflow for 16S rRNA sequencing typically involves several key steps:

- **DNA extraction:** Microbial DNA is extracted from fecal or other biological samples.
- **Amplification:** Specific hypervariable regions of the 16S rRNA gene are Amplification: specific hypervariable regions of the 16S rRNA gene are targeted with **broad-range primers**; primer–template mismatches and region choice can differentially capture taxa, introducing representation bias that must be considered (Abellan-Schneyder et al., 2021), in the representation of certain bacterial species, depending on the target region selected and the reference database used (Abellan-Schneyder et al., 2021).
- **Sequencing:** High-throughput sequencing platforms such as Illumina MiSeq are used to sequence the amplified regions.
- **Bioinformatic analysis:** The resulting sequencing data are processed using bioinformatic pipelines that include quality control, clustering of Operational Taxonomic Units (OTUs) or inference of Amplicon Sequence Variants (ASVs), and taxonomic classification using reference databases such as Greengenes, SILVA, or the Ribosomal Database Project (RDP) (Patel, 2001). To enrich the practical applicability of this review, we have included a brief discussion on bioinformatic pipeline selection. Multiple studies have shown that although pipelines such as QIIME2, DADA2, and mothur may differ in sensitivity or richness estimates, they generally yield consistent microbial diversity and compositional profiles when applied to the same data. For instance, Ducarmon et al. reported pipeline-dependent variation in observed richness but compositional coherence across diverse sample types (Ducarmon et al., 2020), while a multisite study of gastric microbiomes confirmed reproducible diversity and abundance patterns across QIIME2, DADA2, and mothur analyses conducted by independent groups using the same data (Lehr et al., 2025). These observations highlight the importance of careful pipeline selection and transparent reporting practices to ensure reliable and comparable results in microbiome research.

### Shotgun metagenomics

Shotgun metagenomic sequencing provides a more comprehensive view of the microbiome compared to targeted approaches such as 16S rRNA gene sequencing, as it captures all DNA present in a sample, including bacterial, viral, fungal, and archaeal genomes. By sequencing all genetic material, shotgun metagenomics offers functional insights into metabolic pathways and the potential activities of the microbial community. This method also facilitates the discovery of novel organisms and genes that may not be detectable using targeted sequencing strategies.

Shotgun sequencing enables quantitative analysis of microbial communities, allowing for the determination of the relative abundance of different species and genes (Hillmann et al., 2018). The procedure includes several critical steps:

- **DNA extraction:** Genomic DNA is extracted from the sample of interest.
- **Fragmentation:** The extracted DNA is randomly fragmented into smaller pieces, typically 100 to 800 base pairs in length.
- **Library preparation:** The fragments are then prepared for sequencing by adding adapters to their ends. These adapters contain sequences required for binding to the sequencing platform and initiating the sequencing reaction.
- **Sequencing:** The prepared library is loaded onto a sequencing platform (e.g., Illumina), where each DNA fragment is sequenced in parallel, producing short reads.
- **Data assembly:** The resulting short reads are assembled into longer contiguous sequences by aligning overlapping regions. For single-genome sequencing, these reads are assembled into contigs and eventually into a complete genome. In metagenomic samples, reads are assembled to reconstruct the genomes of individual species within the community.
- **Data analysis:** The assembled sequences are analyzed to identify genetic elements and functional genes, and to annotate microbial genomes. In metagenomic studies, this includes taxonomic classification to determine community composition and functional analysis to explore the roles of various genes (Ranjan et al., 2016).

### Shallow metagenomics and methodological limitations

Shallow metagenomics is a cost-effective alternative to both full shotgun sequencing and targeted 16S rRNA amplicon sequencing. This low-depth sequencing technique analyzes limited portions of DNA rather than the entire genome. Although it provides less detailed and accurate information compared to shotgun or full 16S rRNA sequencing, it still offers a general overview of microbial composition—including multiple genomic regions such as 16S rRNA.

The workflow is similar to that of shotgun sequencing, but with significantly lower read depth (Xu et al., 2021).

However, none of these three methods—particularly in the context of fecal analysis—can provide information about the mucosal microbial compartment or the physiological daily fluctuations of the microbiota. It is important to recognize that fecal analysis is performed on luminal content, which is spontaneously expelled.

The main advantages of these approaches are their non-invasiveness and virtually unlimited analytical repeatability. However, the key limitation lies in the inaccessibility of the mucosal compartment, which is notably different from the luminal one—especially in terms of lower richness and a higher proportion of Proteobacteria (Vaga et al., 2020).

Additionally, the inability of these tests to evaluate diurnal physiological fluctuations limits the interpretive value of the data. Assigning immunological, metabolic, or functional significance—or inferring mucolytic activity or intestinal permeability—based solely on the presence or absence of specific bacterial groups is currently unfounded, as it lacks validation from robust studies.

Even more problematic is the assignment of numerical values, even approximate ones, to the potential production of short-chain fatty acids (SCFAs) such as acetate, propionate, and butyrate, based on the taxa identified. Notably, such functional assessments can only be performed using cadaveric models that allow for direct quantification of microbial activity.

### Describable parameters

Despite the limitations discussed above, certain parameters can be validly assessed through microbiota analysis:

- **Biodiversity:** This refers to the variety and abundance of bacterial species present in a given sample. It is quantified using indicators such as the number of Operational Taxonomic Units (OTUs), the Chao1 Index, and the Shannon Index. These metrics provide insight into the richness and evenness of microbial populations.
- **Taxonomy**: Taxonomic classification organizes microorganisms hierarchically, from phylum to species level. In shotgun metagenomics, this can extend further to strain-level identification and metabolomic characteristics, offering high- resolution data on microbial identity.
- **Enterotypes:** These are distinct clusters of microbiota configurations that reflect and may influence gut health. The primary types include Bacteroides 1, Bacteroides 2, Prevotella, and Ruminococcus. Each enterotype is associated with specific dietary patterns and potential health implications.
- **Microbial ratios and associations:** Specific relationships between microbial groups can provide additional insights, including:
  - Firmicutes/Bacteroidetes ratio
  - Gram-positive/Gram-negative ratio
  - Prevotella/Bacteroides ratio
  - Fusobacterium nucleatum/Faecalibacterium prausnitzii ratio

These parameters can serve as useful comparative tools for evaluating changes in microbiota composition but should be interpreted with caution and in conjunction with clinical context (Di Pierro et al., 2024a).

### Quality control and data preprocessing

To ensure accurate interpretation and a more reliable understanding of sequencing results, the implementation of positive and negative controls is essential:

- **Positive controls**, such as mock communities (synthetic bacterial communities with known composition) and spiked-in strains (artificially added bacteria), are used to assess the accuracy and sensitivity of the analytical workflow.
- **Negative controls** are crucial for detecting potential contamination during DNA extraction or library preparation steps. These controls help to validate the integrity of the entire sequencing process.

Additionally, the use of bioinformatic filtering tools, such as the DADA2 platform, enhances sequence quality by removing errors and artifacts.

Preprocessing of sequencing data is a critical step to ensure result reliability. It involves:

- Removing low-quality reads;
- Assembling DNA fragments; and
- Generating more accurate and comprehensive microbial profiles.

However, it is important to acknowledge inherent limitations in the methodologies described—particularly in fecal sample analysis—due to the absence of information about the mucosal compartment, which plays a distinct and significant role in host– microbiota interactions.

### Standardized testing procedure

A standardized procedure is essential to ensure reliable results in microbiota analysis. It requires well-defined protocols, beginning with sample collection and continuing through to final data analysis:

1. **Fecal sample collection and preservation:** The fecal sample must be collected using a laboratory-provided kit that includes a sterile container to prevent contamination, a device for proper sample handling, and a chemical preservative to stabilize bacterial DNA. Kits should be supplied to the patient with clear, detailed instructions for use. Each sample must be accurately labeled to ensure correct patient identification and time of collection. Samples should be packaged in accordance with safety standards for both transport and disposal, minimizing risks to handling personnel. Standardization of the collection process is crucial to prevent microbial DNA degradation. The use of chemical stabilizers and cooled transport systems is recommended to maintain sample quality until analysis (Ilett et al., 2019).
2. **Use of the Bristol Stool Chart:** The quality of the fecal sample can significantly influence microbiome results. The use of the Bristol Stool Chart allows for classification of stool characteristics (shape and consistency), providing important contextual information for interpreting results. This becomes an essential element for meaningful clinical interpretation (Vandeputte et al., 2016).
3. **DNA extraction:** Upon receipt of the fecal sample, DNA is extracted using validated techniques to prevent external contamination. From DNA extraction to sequencing, the entire process must follow standardized protocols to ensure high-quality data. Extraction must avoid contamination and loss, and subsequent DNA amplification—via 16S rRNA gene sequencing or shotgun metagenomics—should be performed uniformly to obtain a comprehensive profile of microbial diversity. Sequencing quality must be monitored to ensure that the data are accurate and complete for downstream analysis (Tourlousse et al., 2021).
4. **Sequencing and analysis:** The extracted DNA is amplified using polymerase chain reaction (PCR) to generate abundant copies of the target genes (typically 16S rRNA). These genes are sequenced using Next Generation Sequencing (NGS) platforms, which provide high-resolution data and enable the identification of a broad spectrum of microbial species in the sample. Sequencing data are then processed using bioinformatic software that performs sequence alignment, taxonomic assignment (identification of bacterial species), and statistical analysis to determine microbial diversity and richness. Results are compared with reference databases to determine microbial composition. All laboratory equipment must be calibrated and certified to ensure operational accuracy. Bioinformatics software must be updated and validated to guarantee reliable outcomes. It is also essential to specify the taxonomic database used for assigning microbial sequences, as the reliability of results depends heavily on the quality of the database, which should be regularly validated and updated by the scientific community (Porcari et al., 2025).
5. **Clinical report:** Once analysis is complete, the laboratory issues a detailed report to the patient or healthcare provider. The report may include graphical representations such as bar plots and box plots to aid in data interpretation, along with all necessary explanatory notes and appropriate bibliographic references for clinical context.

**Table 1:** Direct-to-consumer microbiota analysis: primary and fundamental requirements.

Topic	Requirements
Collection, transport and storage methods	All methods must be validated, including collection tools, preservatives that inactivate all microbes (while preserving DNA), and storage at –80°C.
DNA extraction kits	Must be validated and diversified for both fecal and vaginal samples.
Amplification primers	Must be documented as capable of amplifying all detectable microbial DNA in the sample.
Analytical procedures	The most robust scientific data have been published using Illumina-based procedures.
Analytical controls	In addition to negative controls, a positive control community (mock community*) must be used.
Filtering	Default settings of the DADA2 platform should be applied, with base quality scores above 75%.
Sequence homology	100%.
Nomenclature database	A database must be selected in which taxonomic names and reads are updated every 12–18 months.
Reference database	A database^ with potentially thousands of healthy individuals should be used to calculate differences from the analyzed sample.
Co-occurrence analysis	This analysis reveals the bacterial network within the microbiota and may suggest potential probiotic approaches.

## Clinical interpretation and utility of microbiota testing

### Analytical parameters

Clinical interpretation must rely on validated parameters, including:

- **Alpha and beta diversity:** Alpha diversity metrics—such as richness and evenness—should always be calculated in microbiome analysis, as they provide ecological insight into the complexity and structure of the microbial ecosystem, which may be associated with clinical response. However, further studies are required to clarify its exact role in clinical practice (The Integrative et al., 2019). Beta diversity, measuring the ecological similarity between microbial communities, should be included when comparing longitudinal samples, multiple anatomical sites, or pathological versus healthy conditions. Its clinical significance, however, still needs to be better defined (Pascal et al., 2017; Pickard et al., 2017).
- **Taxonomic composition:** Taxa should be identified at all possible levels—from phylum to genus or species using amplicon sequencing, and to species or strain level using whole genome sequencing (WGS), with their relative contribution estimated across the entire community (Knight et al., 2018). Marker gene-based mapping or *de novo* assembly with reconstruction of metagenome-assembled genomes (MAGs) can be used in WGS workflows (Quince et al., 2017; Blanco-Míguez et al., 2023). Importantly, comparison with a well-matched healthy control group should be included to contextualize diversity and composition metrics. A major challenge lies in defining what constitutes an appropriate control, which must be reasonably comparable to the test subject, an open issue yet to be resolved.
- **Key bacterial relationships:** Reporting of the Firmicutes-to-Bacteroidetes ratio is discouraged in microbiome testing, as current evidence does not support the use of such indices as diagnostic dysbiosis markers. High-level phylum descriptors may fail to capture the complexity of microbiome variation and can lead to misleading interpretations. For example, high relative abundance of *Bacteroides* spp. may indicate either a healthy or an altered ecosystem depending on the context (Knights et al., 2014).

While various dysbiosis indices have been proposed (Gupta et al., 2020; Gacesa et al., 2022), no consensus definition exists, making dysbiosis an unsuitable concept for routine clinical use at present. If such metrics are reported, it is essential that they are appropriately contextualized and accompanied by clear disclaimers.

### Clinical applications

Microbiome analysis is not intended as a diagnostic tool for specific diseases, but rather to assess the possible influence of the microbiota on an existing pathological condition. In other words, it can indicate whether the microbiota plays a role in disease progression, but it does not directly diagnose the condition. Furthermore, there is no universally accepted definition of eubiosis to date. Everyone has a unique microbial composition influenced by genetic, environmental, and dietary factors.

Interpreting the microbiota composition may be a valuable strategy for managing and monitoring chronic diseases, metabolic disorders, neurodegenerative diseases, and gastrointestinal conditions. It also enables personalized therapeutic approaches, such as the targeted prescription of probiotics and customized dietary strategies to restore and maintain microbial balance.

Numerous studies have explored the use of probiotics in different therapeutic contexts, including as an adjuvant treatment for *Helicobacter pylori* eradication (Di Pierro et al., 2020b), in combination with conventional therapy for diverticular disease (Di Pierro et al., 2020a), and in the management of respiratory diseases (Di Pierro et al., 2022).

Another potential application is in the field of sports performance optimization. Scientific evidence shows that intense physical exercise—such as swimming, rowing, cycling, or triathlon—induces physiological adaptations that impact not only performance but also metabolic, immune, and intestinal health (MacKinnon, 2000; Costa et al., 2017; Marttinen et al., 2020). Athletes, due to high training loads, often experience gastrointestinal symptoms such as bloating, cramps, nausea, flatulence, abdominal pain, altered bowel movements, leaky gut syndrome, and vomiting. These symptoms are associated with digestive tract inflammation, increased permeability, and microbial imbalance, including overgrowth of *Prevotella* spp (Bonomini-Gnutzmann et al., 2022). and a decreased Firmicutes/Bacteroidetes ratio (Scheiman et al., 2019). This dysbiosis can promote bacterial translocation and systemic inflammation, adversely affecting both athletic performance and gut health (Dalton et al., 2019). Some studies suggest that probiotic supplementation (e.g., *Lactobacillus* and *Bifidobacterium*), combined with dietary strategies and specific nutraceuticals, may reduce symptoms and improve performance (Shing et al., 2014; Bertuccioli et al., 2024). However, further studies are needed to fully validate these findings.

Gut microbiota analysis may also support early colorectal cancer screening. Literature reports describe cases in which high levels of *Fusobacterium nucleatum* in patients with gastrointestinal symptoms contributed to personalized treatment decisions with clinical benefit. Combining microbiome analysis with traditional tools such as fecal occult blood tests could enhance early detection, diagnosis, and prevention strategies (Di Pierro et al., 2024b).

Finally, in autism spectrum disorders (ASD) with gastrointestinal involvement, the microbiota appears to play a critical role in clinical management. Emerging literature suggests that analyzing the microbiota may assist in developing therapeutic strategies, where dietary and microbiome-targeted interventions may improve patient outcomes. Further studies are needed to clarify the mechanisms involved (Bertuccioli et al., 2022).

### Limitations and warnings

Despite its potential, microbiota testing must be interpreted with caution, acknowledging several limitations:

- **Interindividual variability:** Microbiota composition is dynamic and influenced by diet, stress, medications, and other factors. It varies significantly across individuals, making it difficult to establish a universal reference or “ideal profile”.
- **Unvalidated interventions:** Microbiota testing should be viewed as a complementary tool within a comprehensive clinical framework and should be interpreted by qualified professionals to avoid misdiagnosis or inappropriate interventions. It should not be used to diagnose disease but rather to support cases where conventional methods have failed to yield satisfactory results.

## Challenges and future directions

Microbiota testing holds significant promise, but scientific evidence remains limited, though expanding. A major obstacle to wider adoption is the lack of analytical standardization, which compromises data comparability across laboratories. Another critical gap lies in professional education: intestinal microbiota is often overlooked or minimally addressed in medical curricula and specialty training programs. As a result, many healthcare professionals lack the tools to interpret microbiota tests or to intervene therapeutically.

Furthermore, both high-quality and unreliable tests are currently available on the market, often indistinguishable to non-expert users.

## Future goals

Future efforts should focus on:

- Promoting ongoing training programs for healthcare professionals on the use and interpretation of microbiota testing.
- Validating clinical utility through stronger scientific research and more rigorous methodological frameworks.
- Improving affordability by reducing costs without compromising analytical quality.

## Conclusions

Microbiota analysis offers enormous potential for personalized medicine, but a rigorous scientific approach must be maintained. We also acknowledge that the choice of bioinformatics pipelines and reference databases can materially influence estimates and, at times, clinical interpretation. To mitigate this, we recommend explicit reporting of software and database releases, key parameters and their rationale, and, where feasible, simple sensitivity checks to confirm the robustness of main findings. A pragmatic minimal reporting checklist is provided (**Table 1**).

The Microbiota International Clinical Society (MICS) advocates for increased evidence-based standardization, continuous research, and educational programs for clinicians to ensure that microbiota testing can meaningfully contribute to improved clinical care and patient well-being.
